# Phloretin inhibits glucose transport and reduces inflammation in human retinal pigment epithelial cells

**DOI:** 10.1007/s11010-022-04504-2

**Published:** 2022-06-30

**Authors:** Maria Hytti, Johanna Ruuth, Iiris Kanerva, Niina Bhattarai, Maria L. Pedersen, Carsten U. Nielsen, Anu Kauppinen

**Affiliations:** 1 School of Pharmacy, Department of Health Sciences, University of Eastern Finland, Yliopistonranta 1 C, 70210 Kuopio, Finland; 2 School of Medicine, Department of Health Sciences, University of Eastern Finland, Yliopistonranta 1 C, 70210 Kuopio, Finland; 3 Department of Physics, Chemistry and Pharmacy, University of Southern Denmark, Campusvej 55, 5230 Odense, Denmark

**Keywords:** Retinal degeneration, Inflammation, Phloretin, Glucose transport, Nuclear factor erythroid 2-related factor 2 (Nrf2)

## Abstract

During age-related macular degeneration (AMD), chronic inflammatory processes, possibly fueled by high glucose levels, cause a breakdown of the retinal pigment epithelium (RPE), leading to vision loss. Phloretin, a natural dihydroxychalcone found in apples, targets several anti-inflammatory signaling pathways and effectively inhibits transporter-mediated glucose uptake. It could potentially prevent inflammation and cell death of RPE cells through either direct regulation of inflammatory signaling pathways or through amelioration of high glucose levels. To test this hypothesis, ARPE-19 cells were incubated with or without phloretin for 1 h before exposure to lipopolysaccharide (LPS). Cell viability and the release of pro-inflammatory cytokines interleukin 6 (IL-6), IL-8 and vascular endothelial growth factor (VEGF) were measured. Glucose uptake was studied using isotope uptake studies. The nuclear levels of nuclear factor erythroid 2-related factor 2 (Nrf2) were determined alongside the phosphorylation levels of mitogen-activated protein kinases. Phloretin pretreatment reduced the LPS-induced release of IL-6 and IL-8 as well as VEGF. Phloretin increased intracellular levels of reactive oxygen species and nuclear translocation of Nrf2. It also inhibited glucose uptake into ARPE-19 cells and the phosphorylation of Jun-activated kinase (JNK). Subsequent studies revealed that Nrf2, but not the inhibition of glucose uptake or JNK phosphorylation, was the main pathway of phloretin’s anti-inflammatory activities. Phloretin was robustly anti-inflammatory in RPE cells and reduced IL-8 secretion via activation of Nrf2 but the evaluation of its potential in the treatment or prevention of AMD requires further studies.

**Supplementary Information:**

The online version contains supplementary material available at 10.1007/s11010-022-04504-2.

## Introduction

Phloretin [3-(4-hydroxyphenyl)-1-(2,4,6-trihydroxyphenyl)-1-propanone] is a naturally occurring dihydroxychalcone found in several fruits, e.g., apples and pears [1, 2]. In vitro studies have revealed phloretin to be anti-inflammatory, anti-cancerous, anti-bacterial, and an antioxidant as well as a scavenger of electrophilic compounds [1–7]. Phloretin has been proposed to be the facilitator of the beneficial effects of apple consumption (reviewed in [8]). The addition of apples or phloretin to the diet decreased intestinal inflammation in rodents and, In vitro, phloretin reduced the secretion of pro-inflammatory cytokines interleukin-8 (IL-8) and monocyte chemoattractant protein-1 (MCP-1) in a colon carcinoma cell line [6, 7].

Phloretin appears to exert its anti-inflammatory properties through multiple pathways, which can vary depending on cell model and experimental conditions [7]. One well-studied effect of phloretin is the inhibition of glucose transporter 1 (GLUT1) and GLUT 2 [9–11]. It has been demonstrated that phloretin inhibits glucose transport across the bovine retinal pigment epithelium (RPE) by 45.5% and that it dose dependently prevents the formation of advanced glycation end products (AGEs) which activate inflammatory processes [7, 12]. Additionally, a part of the phloretin’s anti-inflammatory effects has been shown to be transcription factor mediated as phloretin has been found to downregulate the activity of nuclear factor kappa-light-chain enhancer of activated B cells (NF-κB), and the mitogen-activated protein kinases (MAPKs) JNK, p38, and the extracellular signal-regulated kinase (ERK) 1/2 [1, 2, 5, 6, 13]. Phloretin was also shown to increase the nuclear translocation of nuclear factor erythroid 2-related factor 2 (Nrf2) in methylglyoxal-treated RPE cells and in the lung tissue of mice suffering from LPS-induced acute lung injury [5, 14].

RPE cell inflammation plays a key role in the development of dry age-related macular degeneration (AMD), a disease that leads to the irreversible loss of central vision (reviewed in [15]). As the average age of the world population continues to increase, the number of AMD cases is steadily rising. Currently, there is no available treatment option for dry AMD, the most prominent subtype of the disease. Chronic inflammation and increased oxidative stress are known to be involved in RPE cell death and dysfunction, which underly the retinal atrophy observed in advanced dry AMD [15]. The loss of RPE cells represents the first step in the loss of vision associated with AMD, followed by the degeneration of photoreceptors, which rely on the RPE for nutrient supply and waste disposal.

Glucose is rapidly depleted in the retina, which causes a glucose gradient that is counteracted by transporter-mediated diffusion across the RPE, leading to a high glucose content inside RPE cells [12]. High glucose conditions have been shown to promote increased oxidative stress, inflammation, migration, and cell death in RPE cells [16–18]. In addition, GLUT1 transporter levels have been shown to be upregulated during aging and to contribute to increased chronic inflammation [10].

These effects raise the intriguing possibility that phloretin could decrease RPE cell inflammation via a reduction of high intracellular glucose levels and AGE formation, while simultaneously regulating transcription factor-mediated inflammatory pathways independent of glucose availability. These effects could prevent RPE cell loss and slow the progression of AMD. Here, we utilized an in vitro model of lipopolysaccharide (LPS)-induced inflammation to examine the effects of phloretin pretreatment on RPE cell inflammation and to illuminate the pathways involved.

## Methods and materials

### Materials

D-[^14^C(U)]-Glucose (2.9 mCi/mmol), [^14^C(U)]-Methyl-α-D-glucopyranoside ([^14^C]-α-MDG; 250 mCi/mmol), and Ultima Gold™ were purchased from Perkin Elmer (Skovlunde, Denmark). Calcium chloride dihydrate (CaCl_2_·2H_2_O), magnesium chloride (MgCl_2_·6H_2_O), magnesium sulfate (MgSO_4_·6H_2_O), potassium chloride (KCl), potassium phosphate monobasic (KH_2_PO_4_), potassium phosphate dibasic (K_2_HPO_4_), sodium chloride (NaCl), sodium phosphate dibasic (Na_2_HPO_4_·7H_2_O), sodium bicarbonate (NaHCO_3_), choline chloride (C_5_H_14_ClNO), ethylenediamine tetraacetic acid (EDTA), ethylene glycol tetraacetic acid (EGTA), phloretin (PHL), phloridzin (PHZ), dimethyl sulfoxide (DMSO), 4-(2-hydroxyethyl)-1-piperazineethanesulfonic acid (HEPES), phosSTOP, 3-(4,5-dimethyldiazol-2-yl)-2,5-diphenyltetrazolium bromide (MTT), Ponceau S, and lipopolysaccharides from Escherichia coli O111:B4 (LPS) were purchased from Sigma-Aldrich (Merck KGaA, Darmstadt, Germany). Cell culture medium and all supplements for glucose uptake studies were purchased from Sigma-Aldrich (Merck KGaA, Darmstadt, Germany). Cell culture media, 0.25% Trypsin–EDTA and fetal bovine serum (FBS) for all other cell experiments were purchased from Thermo Fisher Scientific (Waltham, MA, USA), while the supplements l-glutamine and penicillin/streptomycin for these experiments were purchased from Lonza (Basel, Switzerland). Dulbecco’s phosphate-buffered saline (DPBS), dl-Dithiothreitol (DTT), RIPA buffer, Mammalian protein extraction reagent (MPER), Tween-20, 2',7'-dichlorodihydrofluorescein diacetate (H_2_DCFDA), and Novex™ Goat anti-rabbit IgG HRP-linked secondary antibody (A16104) were purchased from Thermo Fisher Scientific. Amersham nitrocellulose membrane, Amersham ECL Mouse IgG, HRP-linked secondary antibody (NA931), and Amersham Hyperfilm ECL were from GE Healthcare Life Sciences (Chicago, IL, USA). All primary antibodies were from Santa Cruz Biotechnology (Dallas, TX, USA), and the Immobilon™ Western Chemiluminescent HRP Substrate (ECL) was purchased from Merck Millipore (Billerica, MA, USA). All reagents used were either for Life Science, tested for molecular biology/cell culture or Reagent grade. Manufacturers’ information for specific kits is indicated where they were used below.

### Cell culture conditions

ARPE-19 cells (ATCC Cat# CRL-2302, RRID:CVCL_0145) were obtained from American Type Culture Collection (ATCC; Manassas, VA, USA) and used for experiments between passage numbers 25 and 35. The cells were maintained in Dulbecco’s Modified Eagle’s Medium/Nutrient Mixture F-12 Ham (DMEM/F12) containing 100 units/ml penicillin, 100 µg/ml streptomycin, 2 mM l-glutamine, and 10% FBS. Cell culture media used in the glucose uptake inhibition studies were supplemented with 1.95 g/L sodium bicarbonate. Cells were kept in a + 37 °C humidified incubator in an atmosphere enriched with 5% CO_2_. ARPE-19 cells were allowed to grow to 100% confluence and sub-cultured once a week, using 0.25% Trypsin–EDTA, with medium changes every other day. In the experiments, cells were plated on tissue culture-treated 10 cm dishes, 12-well, or 96-well plates, and incubated for 72 h until completely confluent. The medium was changed to serum-free DMEM/F-12 (17.5 mM d-glucose), DMEM high glucose (HG, 25 mM d-glucose), or DMEM without added glucose (noG, 0 mM d-glucose) medium, and cells were incubated with varying concentrations of phloretin (PHL), 10 µM SP600215, sulforaphane (SUL), or equimolar amounts of the vehicle DMSO for 1 h. After 1 h, LPS was added and cells were incubated for an additional 2 h (signaling pathway analysis) or 24 h (release of pro-inflammatory cytokines and cell viability studies) before sample collection and analysis. In the glucose uptake experiments, ARPE-19 cells were seeded onto tissue culture-treated 24-well plates. The cells were cultured for 72 h before experiments were performed. In the experiments with pre-treatments, 10 µg/ml LPS was added for either 24 h (in cell-culture medium) or for 1 h, or concomitantly before the uptake experiments. Phloretin was dissolved in DMSO, and 100 µM of phloretin in 10 mM HEPES in glucose-free HBSS (pH 7.40) solution with the final concentration of 0.1% DMSO was added 1 h, 2 h, or immediately before measuring the cellular uptake of glucose.

### Microscopy

Cells were analyzed under an inverted phase-contrast light microscope (Carl Zeiss AG, Oberkochen, Germany) at the collection time point (24 h post LPS). At least two images were taken per well at the magnification of 80X. Representative images are shown.

### Cell viability assays

The MTT assay was used to assess cellular viability by measuring the metabolic activity of cells, i.e., their capability to transform the MTT salt into formazan. Briefly, MTT salt was added to cell cultures at a final concentration of 0.5 mg/ml. Cells were incubated in the dark for 90 min at + 37 °C, after which the MTT-containing medium was replaced with DMSO. After an additional incubation of 20 min at room temperature, the formazan crystals, dissolved in DMSO, were collected and transferred to a 96-well plate. The optical density of each well was measured at 562 nm, and results were calculated in relation to those of untreated control cells, which were considered to represent 100% viability.

### Enzyme-linked immunosorbent Assays (ELISA)

The levels of IL-6 and IL-8 were determined from cell-culture medium samples collected 24 h after LPS addition using BD OptEIA human Enzyme-linked Immunosorbent Assay (ELISA) kits (BD, Franklin Lakes, NJ, USA). Vascular endothelial growth factor (VEGF) levels were determined from cell-culture medium samples collected 24 h after LPS addition using the human VEGF DuoSet ELISA kit (R&D Systems Inc. Minneapolis, MN, USA). The levels of phosphorylated JNK, CREB, p38, or ERK1/2 were determined from cell lysates collected 2 h after LPS stimulation using PathScan® Sandwich ELISA kits from Cell Signaling Technology (Danvers, MA, USA). Cell lysates were collected on ice and in the presence of phosSTOP in the cell lysis buffer provided with the ELISA kits. The binding affinity of the p65 subunit of NF-κB was measured using the TransAM® NF-κB p65 activation assay (Active Motif, Carlsbad, CA, USA). All ELISAs were performed according to the manufacturer’s instructions.

### Glucose uptake studies in ARPE-19 cells

Two different probes were used to investigate transporter-mediated uptake of glucose in ARPE-19 cells. Glucose is transported into cells via a variety of sodium-dependent glucose transporters (SGLT1 and SGLT2) and sodium-independent transporters (e.g., GLUT1 and GLUT2), while α-MDG is only transported via the sodium-dependent glucose transporter. ARPE-19 cells were equilibrated for 10 min at 37 °C and 220 rpm on an incubating microplate shaker in 10 mM HEPES in glucose-free HBSS adjusted to pH 7.40 ± 0.01 using 0.1 or 1.0 M NaOH. Hanks' Balanced Salt Solution (HBSS) glucose-free buffer consisted of 1.3 mM calcium chloride dihydrate (CaCl_2_·2H_2_O), 0.5 mM magnesium chloride (MgCl_2_·6H_2_O), 0.4 mM magnesium sulfate (MgSO_4_·6H_2_O), 5.3 mM potassium chloride (KCl), 0.4 mM potassium phosphate monobasic (KH_2_PO_4_), 137.9 mM sodium chloride (NaCl), 0.3 mM sodium phosphate dibasic (Na_2_HPO_4_·7H_2_O), and 4.2 mM sodium bicarbonate. In the HBSS glucose- and sodium-free buffers, 137.9 mM sodium chloride (NaCl) and 0.3 mM sodium phosphate dibasic (Na_2_HPO_4_·7H_2_O) were replaced with 137.9 mM choline chloride (C_5_H_14_ClNO) and 0.3 mM potassium phosphate dibasic (K_2_HPO_4_), respectively.

In the glucose uptake experiments, donor solutions of 0.25 μCi/ml [^14^C]-glucose (86.2 µM) in the absence or presence of 0.1 mM phloretin (a GLUT-type inhibitor) (0.1% DMSO), 1.0 mM phloretin (1% DMSO), or 0.01 mM phloridzin (a SGLT-type inhibitor) in the presence or absence of 10 µg/ml LPS were added to the cells. After 5 min incubation on a shaker set to 220 rpm at 37 °C, the solutions were removed. In the α-MDG uptake experiments, donor solutions of 0.5 μCi/ml [^14^C]-α-MDG (2.0 µM) in the absence or presence of 0.1 mM phloretin (0.1% DMSO) or 0.01 mM phloridzin were added to the cells. After 10 min incubation at 220 rpm and 37 °C, the solutions were removed. After the isotope uptake studies, the cells were washed three times with ice-cold glucose-free HBSS and detached using 200 µl 0.5% Trypsin–EDTA solution for at least 20 min at 37 °C. The cell homogenate was transferred to scintillation vials, and 2 ml Ultima Gold scintillation liquid was added. All scintillation vials were then vortexed thoroughly before being analyzed by liquid scintillation counting (LSC; PerkinElmer, Tri-Carb 4910TR). The uptake experiments were also performed in glucose- and sodium-free HBSS.

The initial uptake rate (pmol min^−1^  cm^−2^) of D-[^14^C(U)]-Glucose or [^14^C(U)]-Methyl a-D-glucopyranoside in ARPE-19 cells was calculated as the amount of compound (Q, pmol) accumulated in the cells in a 5 or 10 min period (t, min) across the well area (A, cm^2^). The uptake in pmol was calculated by converting disintegrations per minute (DPM) into pmol using the DPM obtained in donor solutions with known molar concentrations.

### Determination of intracellular reactive oxygen species

H_2_DCFDA was used to determine levels of reactive oxygen species in RPE cells. Briefly, cells were cultured on 96-well plates for 72 h until fully confluent. The medium was changed to serum-free DMEM/F-12 culture medium and cells were treated with 5 µM H_2_DCFDA and 100 µM phloretin, or equimolar amounts of DMSO for 1 h. After 1 h, the wells were washed with DPBS, and LPS was added at the final concentration of 10 µg/ml (in DPBS). Plate fluorescence was measured immediately on a microplate reader measuring fluorescence at 488 nm excitation and 528 nm emission.

### Western blot

Cells cultured and treated on 10 cm dishes were collected into ice-cold fractionation buffer (20 mM HEPES buffer containing 10 mM KCl, 2 mM MgCl_2_, 1 mM EDTA, 1 mM EGTA, and 1 mM DTT with a pH adjusted to 7.4) 2 h after the addition of LPS. After an incubation on ice for 15 min, cells were ruptured by repeated passage through a 26-gauge needle and incubated on ice for an additional 20 min. The nuclear fraction was centrifuged at 720 × g for 5 min and washed in ice-cold fractionation buffer before being centrifuged again at 720 × g for 10 min. The supernatant was discarded, and the nuclear fraction was dissolved in RIPA buffer. The protein concentration of the nuclear fraction was measured using the Bicinchoninic Acid (BCA) Assay (Thermo Fisher Scientific, Waltham, MA, USA). Samples were diluted in distilled water to achieve uniform protein concentrations and stained with a 3 × Laemmli solution. Proteins in the samples were separated on a 10% SDS-PAGE gel at 200 V for 2.5 h before being wet transferred onto a nitrocellulose membrane overnight at 17 V in an ice-cold blotting buffer containing 20% methanol. Membranes were stained with Ponceau S to confirm successful protein transfer and then blocked in a TBS solution containing 0.1% Tween-20 and 5% milk for 2 h at room temperature. The membranes were probed with primary antibodies for Nrf2 (1:1000, sc-722, RRID:AB_2108502), and PCNA (1:1000, sc-56, RRID:AB_628110), overnight at + 4̊ C. Bound primary antibody was detected by addition of either a Novex™ Goat anti-rabbit IgG HRP-linked secondary antibody (1:15 000, A16104, RRID:AB_2534776) or an Amersham ECL Mouse IgG (1:10 000, NA931, RRID:AB_772210) HRP-linked secondary antibody for 2 h at room temperature. HRP-bound antibody was detected on Amersham Hyperfilm after incubation with Chemiluminescent HRP Substrate. Protein bands were scanned and analyzed using ImageJ (US National Institutes of Health, Bethesda, MD, USA; http://imagej.nih.gov.ezproxy.uef.fi:2048/ij/, RRID:SCR_003070).

### Small interfering RNA (siRNA) transfections

siRNA experiments were performed using reverse transfections of Nrf2 siRNA (15,763, Silencer®, Thermo Fisher Scientific) or negative control siRNA (#2, Silencer®, Thermo Fisher Scientific) following the manufacturer’s instructions of the transfection reagent lipofectamine RNAiMAX (Thermo Fisher Scientific). Cells were added to the siRNA complexes at a density of 40,000 cells/0.1 ml/well. Cells were incubated for 24 h until they were fully confluent and then treated with phloretin and LPS as described above. Exposure of cells to siRNAs did not decrease cellular viability (Supplementary Fig. 1).

### Statistical analyses

All experiments were performed in independent cell passages at least three times. Data were combined and expressed as mean ± SEM. Statistical analysis was performed using the GraphPad Prism Software (version 7.01, San Diego, CA, USA, RRID:SCR_002798). Pairwise comparisons of the treatment groups were made using the Mann–Whitney *U-*test, following a Kruskal–Wallis test. Differences between groups were considered statistically significant at *p* < 0.05.

## Results

### Phloretin treatment reduces pro-inflammatory cytokine secretion in stressed retinal pigment epithelial cells

**Fig. 1:**
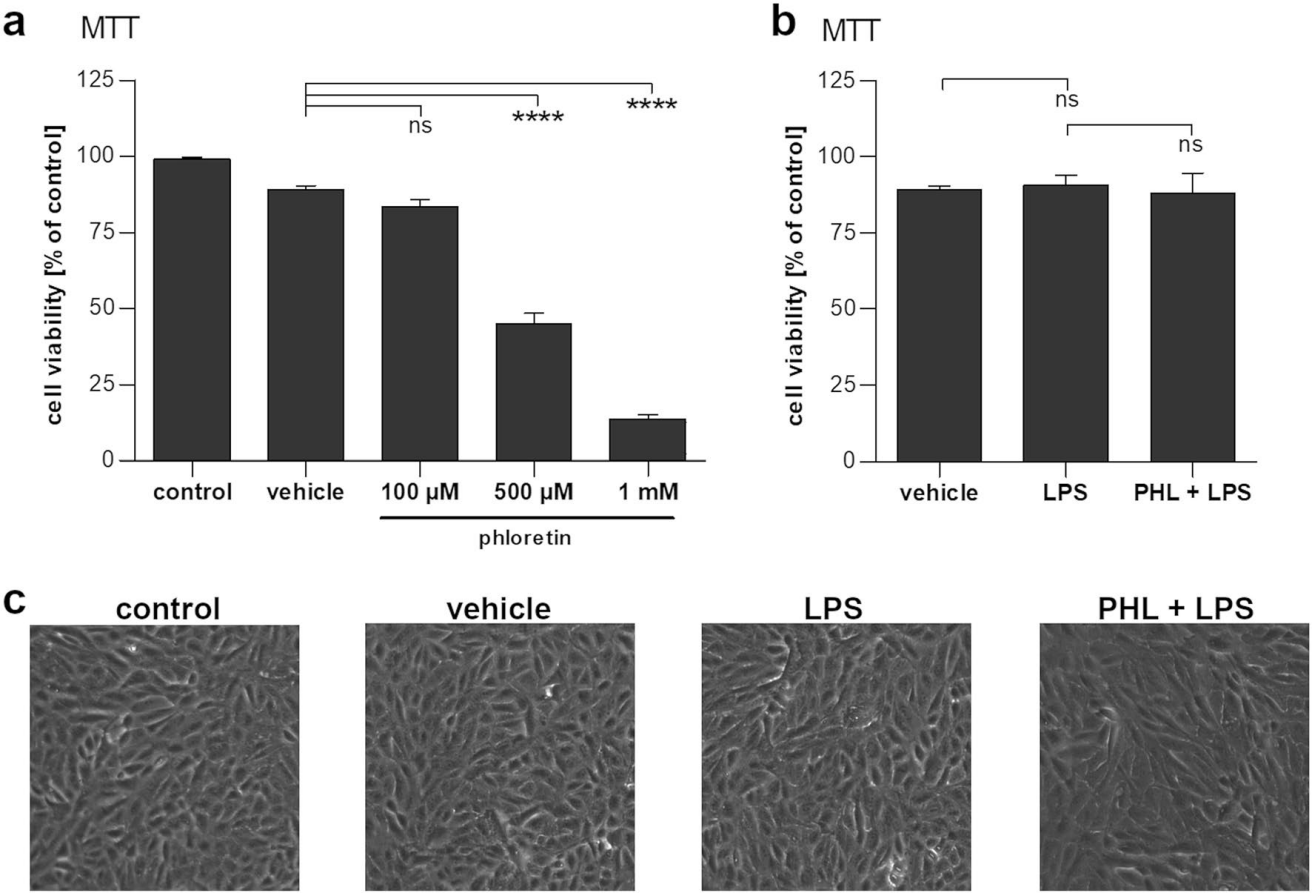
The effect of phloretin on cell viability. **a** Phloretin does not decrease RPE cell viability at the studied concentration of 100 µM. **b** Lipopolysaccharide (LPS) exposure with or without a 1 h pretreatment with 100 µM phloretin (PHL) does not reduce cell viability. **c** Brightfield microscopy images of cells exposed to vehicle or 10 µg/ml LPS with or without 1 h pretreatment with 100 µM phloretin reveal no changes to cellular morphology. Data are combined from three to nine independent experiments with four parallel samples per experiment and are represented as mean ± SEM. Representative microscopy images are shown. PHL—100 µM phloretin, LPS—10 µg/ml LPS, *ns* not statistically significant, ****p < 0.0001, Mann–Whitney U-test

**Fig. 2:**
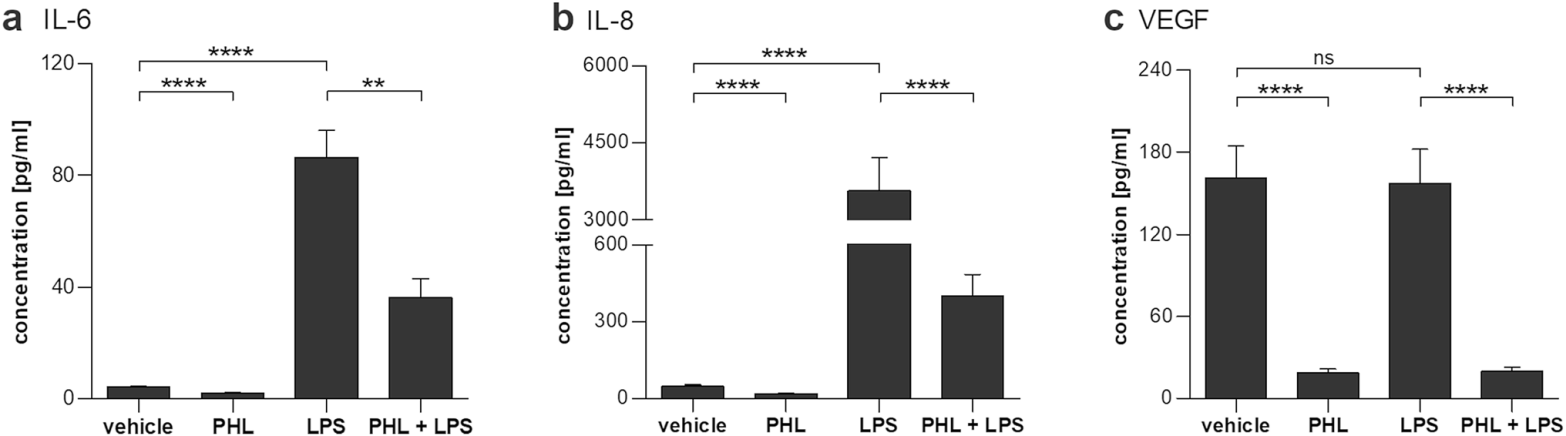
The effect of phloretin on pro-inflammatory cytokine secretion. A 1 h pretreatment with 100 µM phloretin (PHL) greatly reduces 10 mg/ml LPS (LPS)-induced secretion of IL-6 (**a**), IL-8 (**b**) or VEGF (**c**) in ARPE-19 cells. Data are combined from three independent experiments with three or four parallel samples per experiment and are represented as mean ± SEM. *ns* not statistically significant, **p < 0.01, ****p < 0.0001, Mann–Whitney U-test

Phloretin exhibited dose-dependent toxicity at concentrations above 100 µM (Fig. 1a). However, no significant toxicity was observed at a concentration of 100 µM, and all subsequent experiments were performed using this concentration. In order to induce an immune reaction in RPE cells, we exposed the cells to 10 µg/ml LPS; LPS did not affect the viability or appearance of RPE cells (Fig. 1b, c) but caused a robust increase in the secretion of pro-inflammatory cytokines IL-6 and IL-8 (Fig. 2a, b). Phloretin treatment significantly decreased IL-6 and IL-8 levels to 41.8% and 11.2% of LPS-stimulated levels, respectively. LPS had no effect on the secretion of VEGF from ARPE-19 cells, but phloretin reduced VEGF levels in the medium to less than 15% of the levels observed in vehicle control or LPS-treated cells (Fig. 2c). Phloretin decreased the levels of all measured cytokines also in the absence of LPS stimulation (Fig. 2a–c).

### Phloretin effectively inhibits glucose uptake in retinal pigment epithelial cells

**Fig. 3:**
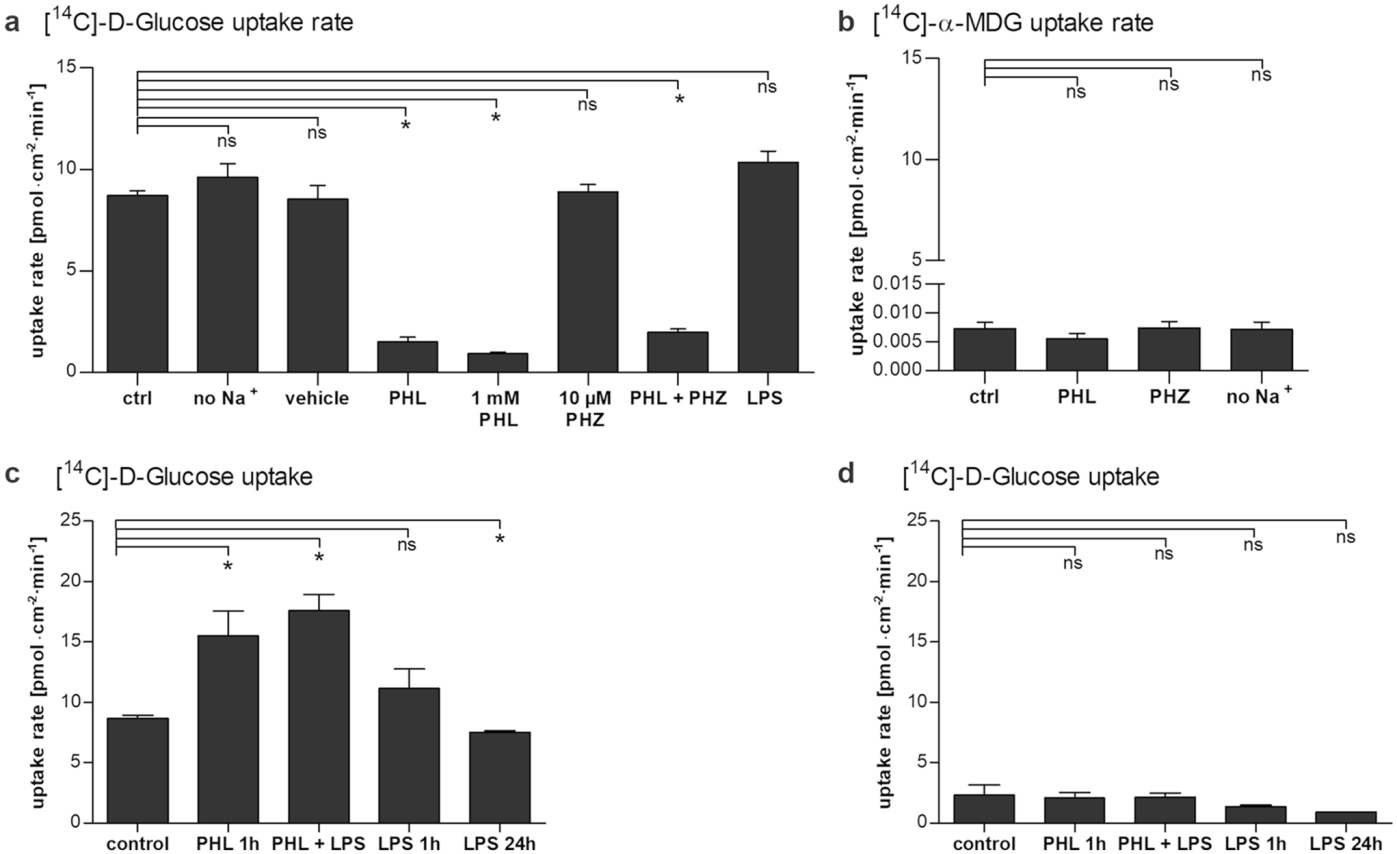
The uptake of [14C]-Glucose and [14C(U)]-Methyl α-D-glucopyranoside ([14C]-α-MDG) by ARPE-19 cells. **a**/**b** The uptake rate of 0.25 μCi/ml [14C]-Glucose (86.2 µM) (**a**) or of 0.5 μCi/ml [14C]-α-MDG (2.0 µM) (**b**) measured over 5 or 10 min, respectively, in the absence or presence of 100 µM phloretin (PHL) or 10 µM phloridzin (PHZ). Phloretin, but not phloridzin significantly reduces the glucose uptake rate. (**c/d**) The uptake rate of 0.25 μCi/ml [14C]-Glucose (86.2 µM) measured for 5 min after 1 h pre-incubation with PHL and LPS, or 24 h incubation with LPS. Glucose uptake was measured in the absence (**c**) or presence of PHL (**d**). Removal of phloretin from culture medium prior to glucose uptake rate measurement leads to an increased uptake of [14C]-Glucose. Data are presented as the mean ± SEM of data from three to five independent experiments. *ns* not statistically significant, *p < 0.05, Mann–Whitney U-test

Phloretin at 100 µM and 1 mM significantly reduced the glucose uptake rate during a 5 min uptake study (Fig. 3a). The uptake of [^14^C]-Glucose in ARPE-19 cells was not affected by the presence of Na^+^-ions, 1% DMSO, or phloridzin, a naturally occurring product found in the bark of pear, apple, and other fruit trees, which is known to be a competitive inhibitor of sodium-glucose co-transporters SGLT1 and SGLT2 (Fig. 3a). A concomitant incubation with 10 µg/ml LPS caused a slight 20% increase of [^14^C]-Glucose uptake; however, this increase was not quite statistically significant (p = 0.0714). The uptake of [^14^C]-α-MDG was not dependent on Na^+^-ions and could not be inhibited by 100 µM phloretin or 0.01 mM phloridzin (Fig. 3b). The DPMs measured for [^14^C]-α-MDG uptake in ARPE-19 cells were not different from background, and hence, the “uptake” represents radioactivity that cannot be washed away. Taken together these results reveal a complete lack of [^14^C]-α-MDG uptake in ARPE-19 cells consistent with the absence of functional SGLT activity.

The uptake of glucose was also measured after pre-incubation of ARPE-19 cells for 1 or 24 h with phloretin or LPS. Phloretin at 100 µM in glucose-free uptake buffer for 1 h prior to measuring glucose uptake, significantly increased the subsequent glucose uptake rate by approximately twofold (Fig. 3c). The combination of 100 µM phloretin and 10 µg/ml LPS also approximately doubled glucose uptake, whereas LPS alone had no effect on glucose uptake (Fig. 3c). The specificity of the increased glucose uptake being due to the removal of phloretin rather than the introduction of glucose was assessed by measuring the uptake after pre-incubation in the presence of 100 µM phloretin (Fig. 3d), where phloretin inhibited the glucose uptake to a similar level independently of the preceding incubation conditions.

### Phloretin’s anti-inflammatory action is independent of glucose concentration in the medium

**Fig. 4:**
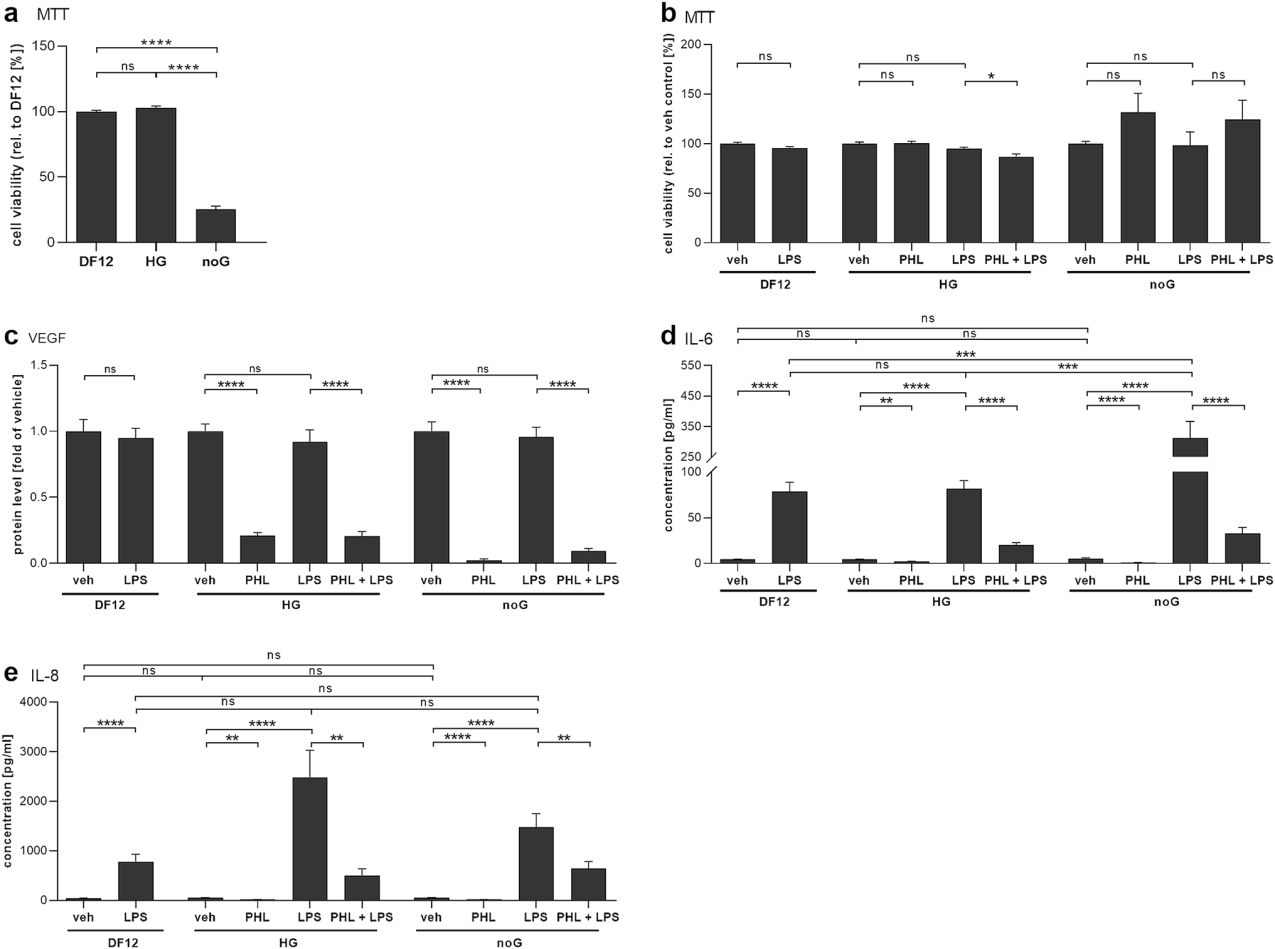
The effect of glucose content in cell-culture medium on cell viability and inflammation. **a** Cellular viability of untreated control cells in no glucose medium (noG) was significantly lowered when compared to high-glucose medium (HG) or normal medium conditions (DF12). **b** 10 µg/ml LPS or 100 µM phloretin (PHL) exposure had little effect on cell viability in the different culture media. **c**–**e** A 1 h pretreatment with 100 µM phloretin (PHL) significantly reduced the 10 µg/ml LPS-induced secretion of VEGF (**c**), IL-6 (**d**) or IL-8 (**e**) in all studied media. Data are combined from three independent experiments with three or four parallels per experiment and are represented as mean ± SEM. *ns* not statistically significant, *p < 0.05, **p < 0.01, ***p < 0.001 ****p < 0.0001, Mann–Whitney U-test

To study the extent by which phloretin’s effects depended on glucose uptake inhibition, experiments in two alternative cell-culture media with either a high glucose concentration (HG) or without glucose (noG) were conducted. RPE cell viability was significantly reduced in the noG medium (Fig. 4a). However, there was no significant change in the relative toxicity of phloretin or LPS in the alternative cell-culture media (Fig. 4b), though the combination of phloretin and LPS was slightly cytotoxic to RPE cells cultured in HG.

Additionally, phloretin decreased the secretion of IL-6, IL-8 and VEGF irrespective of the culture medium (Fig. 4). LPS did not affect VEGF secretion levels in any of the media studied, while phloretin reduced VEGF levels significantly in all media, irrespective of glucose content (Fig. 4c). Despite the lower cell viability in noG medium, cells treated with LPS secreted significantly more IL-6 in the absence of glucose (Fig. 4d). This effect was not observed for IL-8 (Fig. 4e). Phloretin treatment either alone or as a pretreatment added 1 h before LPS significantly reduced IL-6 and IL-8 levels across all culture conditions.

### Phloretin treatment increased intracellular reactive oxygen species and activated Nrf2 but reduced JNK activity

**Fig. 5:**
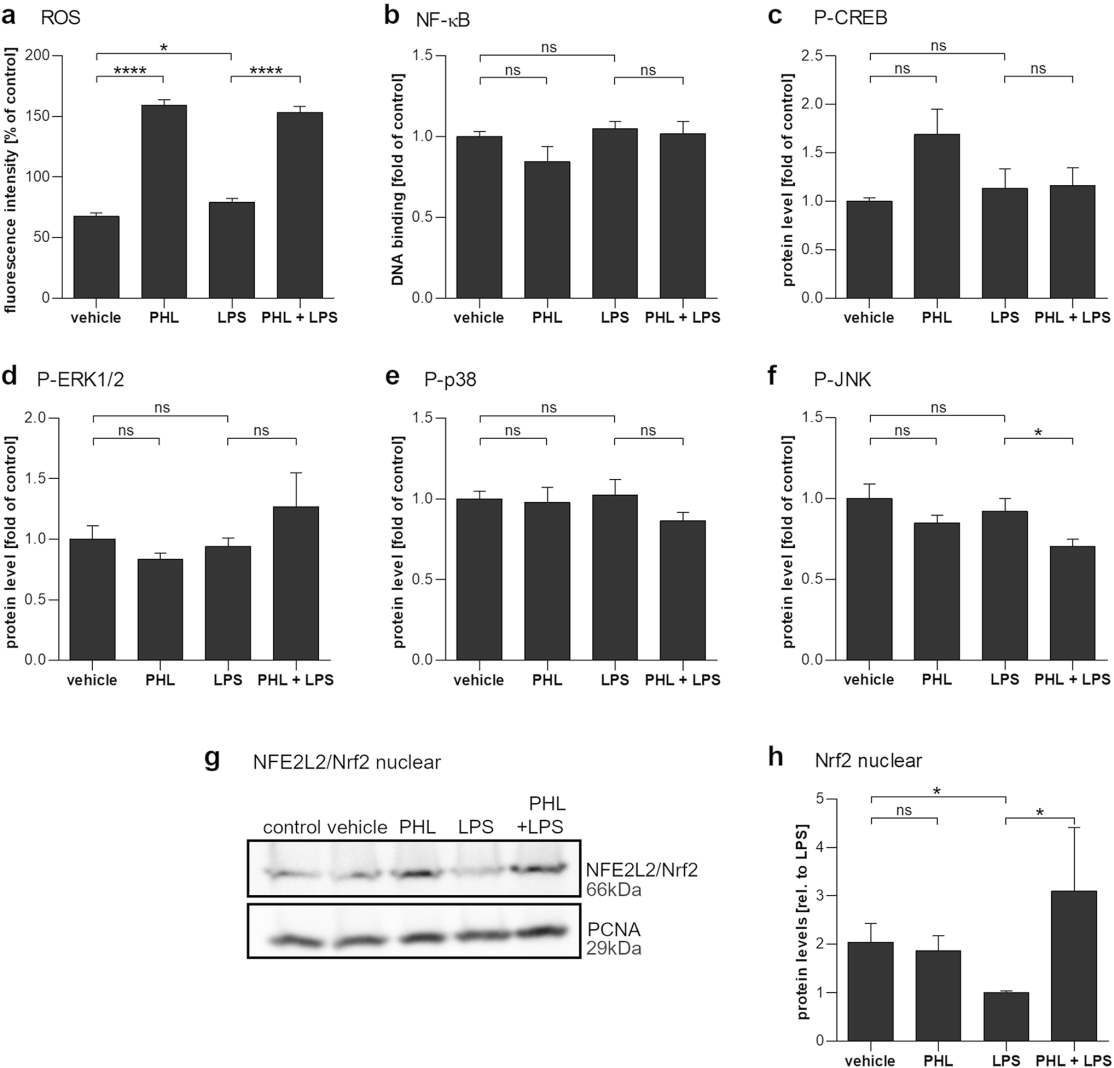
The effect of phloretin and LPS exposure on cellular signaling pathways in ARPE-19 cells. **a** Intracellular ROS levels are significantly increased in cells exposed to 100 µM phloretin (PHL) for 1 h and slightly increased in cells exposed to 10 µg/ml LPS (LPS) for 5 min. **b**–**f** Following a 1 h pretreatment with PHL and a 2 h exposure to LPS, the DNA-binding activity of NF-κB subunit p65 (**b**) or the intracellular levels of phosphorylated CREB (**c**), ERK1/2 (**d**), and p38 (**e**) remained unaffected. The phosphorylation of JNK (**f**) was reduced by cotreatment with 100 µM phloretin and 10 µg/ml LPS. **g**/**h** Phloretin increased the nuclear levels of Nrf2 as determined by Western Blotting (**g**, quantified **h**). Data are combined from three or four independent experiments, with two to four parallel samples per experiment and are represented as mean ± SEM. *ns* not statistically significant, *p < 0.05, ****p < 0.0001, Mann–Whitney U-test

To investigate the underlying signaling pathways, cellular ROS levels, as well as the activity or phosphorylation of selected transcription factors were measured. Exposure of cells to 100 µM phloretin strongly increased intracellular ROS levels (Fig. 5a). Neither LPS nor phloretin treatments influenced the DNA-binding activity of NF-κB subunit p65, nor the phosphorylation of transcription factor CREB or of the MAPKs p38 and ERK1/2 (Fig. 5b–e). However, the 1 h pretreatment of cells with phloretin before LPS exposure significantly prevented the phosphorylation of JNK (Fig. 5f). Furthermore, nuclear levels of Nrf2, which were decreased by LPS stimulation, were significantly elevated after a 1 h pretreatment with phloretin (Fig. 5g, h).

### Nrf2, not JNK, is involved in the anti-inflammatory actions of phloretin

**Fig. 6:**
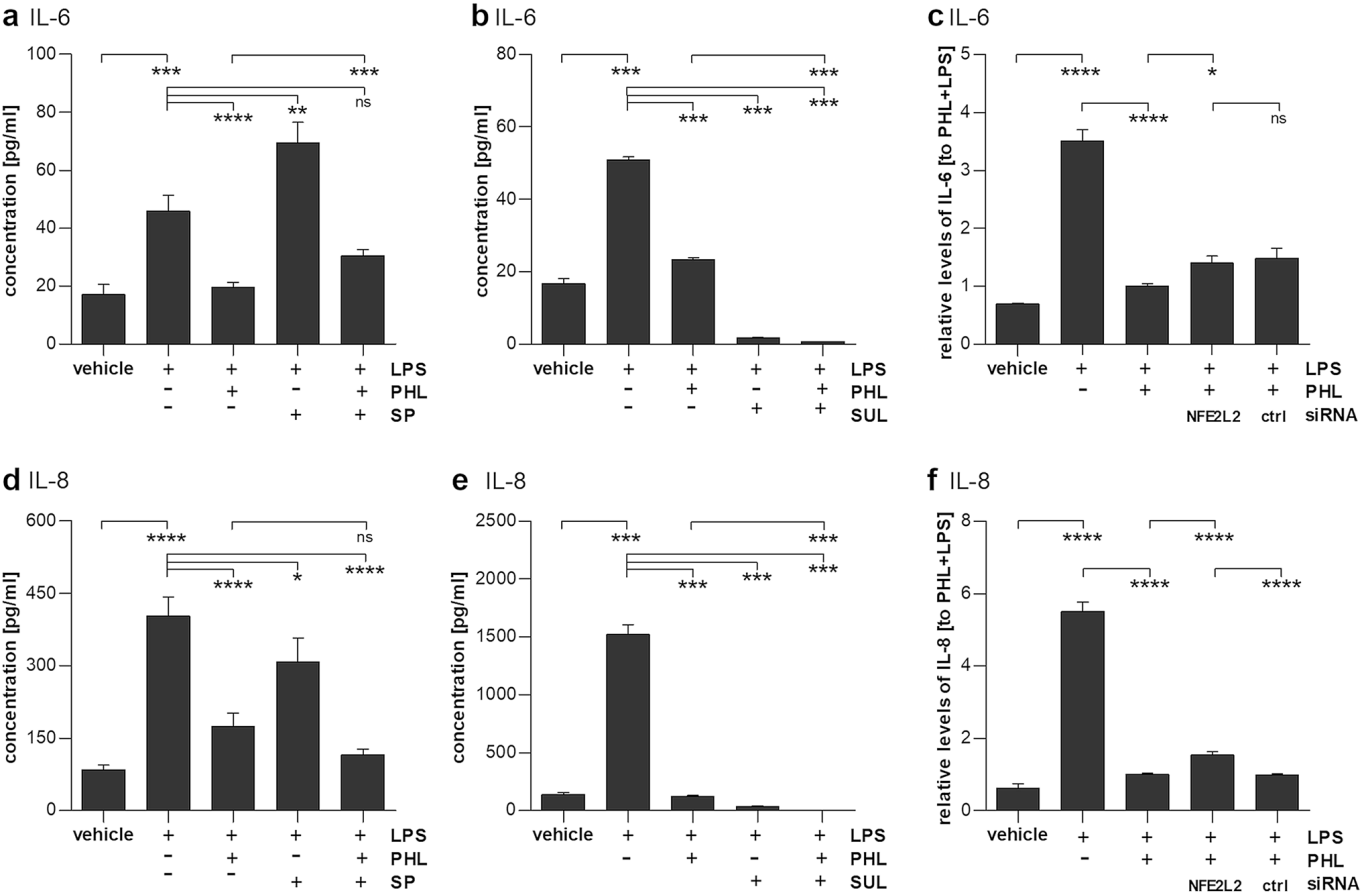
Effect of JNK inhibition or Nrf2 activation and knockdown on LPS-stimulated ARPE-19 cells. **a**/**d** A 1 h pretreatment with the JNK inhibitor SP600215 (SP, 10 µM) increased IL-6 (**a**) and slightly decreased IL-8 (**d**) secretion in 10 µg/ml LPS (LPS)-stimulated ARPE-19 cells. **b**/**e** A 1 h pretreatment with the Nrf2 activator sulforaphane (SUL, 20 µM) significantly decreased the secretion of IL-6 (**b**) or IL-8 (**e**) from LPS-treated RPE cells with or without phloretin (PHL). **c**/**f** Nrf2 knockdown by siRNA transfection before exposure to phloretin (PHL) and LPS increased IL-6 (**c**) or IL-8 (**f**) secretion when compared to phloretin and LPS stimulation alone, but only changes for IL-8 were statistically significantly different to the effect of scrambled control siRNA. Results are combined from three or four independent experiments with three or four parallel samples per experiment and are represented as mean ± SEM. *ns* not statistically significant, *p < 0.05, **p < 0.01, ***p < 0.001, ****p < 0.0001, Mann–Whitney U-test

To further evaluate the importance of JNK or Nrf2-related signaling pathways in the anti-inflammatory effects of phloretin, LPS-treated RPE cells were exposed to the JNK inhibitor SP600215 (SP) or the Nrf2 activator sulforaphane (SUL). Pretreatment of RPE cells with the JNK inhibitor SP600215 significantly reduced IL-8 levels in the medium, though not to the same extent as phloretin alone (Fig. 6d). Co-treatment with both SP600215 and phloretin did not decrease IL-8 levels below those observed with the phloretin treatment alone. Conversely, IL-6 levels were increased after JNK inhibition by SP600215 (Fig. 6a). Treatment of RPE cells with the Nrf2 activator sulforaphane reduced both IL-8 and IL-6 levels (Fig. 6b, e). Sulforaphane’s effect was stronger than that of phloretin alone, decreasing IL-6 and IL-8 levels to 3.4% and 2.2% of LPS-induced levels, respectively, in comparison to 46.4% and 7.9% achieved with phloretin alone. The combination of both phloretin and sulforaphane revealed an additive ameliorating effect on the secretion of both IL-8 and IL-6 (Fig. 6b, e). Knockdown of Nrf2 using siRNA partly reversed the effects of phloretin on IL-8 secretion, while an increase in IL-6 secretion after Nrf2 knockdown failed to be statistically significantly different from the effects of a negative control siRNA (Fig. 6c, f).

## Discussion

Here, we show, for the first time, the protective effects of phloretin in human cultured RPE cells against LPS-induced inflammation and elucidate the involved underlying pathways. We present evidence that phloretin is anti-inflammatory in a model of LPS-induced RPE cell inflammation and that it exerts its effects predominantly through the Nrf2 signaling pathway, while also inhibiting JNK phosphorylation.

Precise molecular factors leading to RPE cell death are complex and not completely elucidated but the roles of inflammation, oxidative stress, and decreased intracellular clearance mechanisms have been well documented (reviewed in [15, 19, 20]). GLUT1 transporter levels are upregulated during aging and can contribute to increased chronic inflammation [10]. Furthermore, in high glucose conditions, RPE cells have been shown to suffer from increased oxidative stress, inflammation, and cell death [16–18]. Inhibiting upregulated glucose transport, reducing intracellular hyperglycemia, and downregulating inflammatory responses might represent ways to alleviate RPE cell stress and prevent or delay the onset of AMD.

Here, we found that phloretin was strongly anti-inflammatory and more than halved the secretion of IL-6, IL-8, and VEGF from unstimulated human RPE cells. The exposure of cultured ARPE-19 cells to LPS increased IL-6 and IL-8 secretion by 20- and 74-fold, respectively, while phloretin pretreatment reduced IL-6 and IL-8 levels to 42% or 11.2%, respectively, of those seen in cells exposed to LPS alone. Similar results have been reported by Huang et al. in LPS-stimulated mouse macrophages, as well as in TNFα-stimulated keratinocytes, and IL-1β-stimulated myofibroblasts [5, 7, 21].

In the present study, phloretin significantly reduced the uptake of glucose in ARPE-19 cells to only 17.6% of vehicle-treated control. Glucose uptake was not affected by sodium ions or phloridzin, suggesting that glucose uptake in RPE cells is facilitated by the GLUT-type carriers rather than the sodium-coupled glucose transporter (SGLT). This proposal is supported by the fact that there was no carrier-mediated uptake of [^14^C]-α-MDG, a specific substrate of SGLT1 and SGLT2. Our findings are in line with previous reports that the transport of glucose across the RPE is transporter mediated and efficiently inhibited by phloretin in bovine eyes [12]. Moreover, Takagi and colleagues reported that cultured human RPE cells predominantly express GLUT1, with only traces of GLUT3 and 5 expression [22]. Taken together, our findings suggest that phloretin decreases glucose uptake in ARPE-19 cells by inhibiting GLUT1. Interestingly, phloretin pretreatment in glucose-free conditions and subsequent removal almost doubled the glucose uptake. This could be caused by either depleted intracellular glucose levels increasing the driving force for uptake or an increased recruitment of GLUT1 into the cell membrane during phloretin-induced inhibition of transporter action. However, it appears unlikely that this affected phloretin’s anti-inflammatory properties. When analyzing phloretin’s effects in cell-culture media containing either a high glucose concentration or no glucose, we found that phloretin continued to be strongly anti-inflammatory regardless of the glucose content. There was no trend for either increased or decreased efficacy of phloretin with higher or lower glucose contents in the medium. This suggests that the inhibition of glucose uptake itself is not the primary mechanism behind phloretin’s anti-inflammatory properties.

Phloretin can, however, affect inflammation also by inhibiting the activity of pro-inflammatory signaling pathways. In a murine model of LPS-induced lung injury, phloretin pretreatment significantly reduced pro-inflammatory cytokine secretion by inhibiting p65 and the phosphorylation of MAP kinases p38, ERK1/2, and JNK, while increasing Nrf2 translocation to the nucleus [5]. Several other reports have reported similar findings on the role of NF-kB, MAP kinases, and/or Nrf2 [1, 2, 5, 6, 13, 14, 23–26]. In contrast to these previous studies, phloretin treatment had no effect on the DNA-binding activity of NF-κB subunit p65 or the phosphorylation of p38 or ERK1/2 in our model. However, phloretin reduced the phosphorylation of JNK by 23.9%. Phosphorylated JNK activates the transcription factor AP-1 by phosphorylation of its subunit c-Jun and it facilitates the initiation of DNA binding [27]. IL-6, IL-8, and VEGF all contain an AP-1-binding domain [27–29]. Cheon et al. have described a JNK-dependent decrease in the secretion of pro-inflammatory cytokines after phloretin treatment in keratinocytes [1]. However, in our ARPE-19 cells, treatment with the JNK inhibitor SP600215 increased LPS-induced IL-6 secretion while significantly decreasing only IL-8 levels. Furthermore, the reduction in IL-8 levels after SP600215 treatment was significantly smaller than that seen with phloretin alone, suggesting that inhibition of JNK phosphorylation is not the main effect underlying phloretin’s anti-inflammatory properties.

In line with the previous reports of Andrisse et al., phloretin exposure significantly increased intracellular ROS levels by 93.7% [30]. Moreover, an increased translocation of Nrf2 to the nucleus was observed, which could be in response to the increased amounts of intracellular ROS. This finding was in line with reports by Huang et al. that phloretin increased Nrf2 translocation in LPS-induced lung injury, and by Sampath et al. who observed increased Nrf2 levels in the nucleus of human RPE cells treated with phloretin and exposed to methylglyoxal [5,14]. In our study, nuclear Nrf2 levels were threefold higher after the phloretin pretreatment when compared to cells treated with LPS alone. The well-established activator of Nrf2, sulforaphane, significantly decreased the release of IL-6 and IL-8 in a similar manner to phloretin, and knockdown of Nrf2 using siRNA significantly reduced phloretin’s ameliorating effect on IL-8 secretion. Taken together, this suggests that Nrf2 activation was the primary pathway through which phloretin exerted its anti-inflammatory effects. However, cotreatment with both phloretin and sulforaphane revealed an additive effect on the IL-6 and IL-8 inhibition, which could point to the participation of other signaling pathways that will need to be addressed in future studies.

Phloretin is known to be readily bioavailable and can be measured in serum after the consumption of apple juice. In Wistar rats on a phloretin-enhanced diet, plasma levels reached concentrations of 67 µM 10 h after food ingestion, a value which might be even higher at earlier timepoints, as another study found phloretin levels in plasma that were highest 5 h after food ingestion in beagle dogs [7, 31]. In humans, urinary excretion levels of phloretin are high enough to be used as a biomarker of fruit intake, and it seems plausible that plasma levels could reach levels similar to the 100 µM used in our study, on a phloretin-enriched diet [32]. Dietary phloretin reduced plasma glucose concentrations, AGEs formation, and insulin levels in a high-fat diet-induced obesity mouse model and improved fasting blood glucose levels, glucose tolerance, and insulin sensitivity in type 2 diabetic rats to a similar degree as metformin treatment [33, 34]. Interestingly, fruit intake has also been found to be protective against the development of AMD, and the AREDS food supplement formulation, containing a mixture of high levels of the antioxidants vitamin C and E, and lutein/zeaxanthin complemented by zinc has been proven to slow the progression from the early to intermediate stages of dry AMD, supporting the potential of a diet-based intervention in the treatment of AMD [35, 36]. What is more, the clear reduction of secreted VEGF levels initiated by phloretin treatment might prove beneficial in the treatment or prevention of neovascular AMD. Anti-VEGF therapy is the current gold standard of treatment for patients suffering from advanced wet AMD, which is characterized by an at least partially VEGF-induced growth of new blood vessels into the subretinal space, where bleeding, swelling, and later scarring lead to a rapid loss in central vision [37].

In conclusion, phloretin showed robust anti-inflammatory potential and caused decreased glucose uptake and VEGF secretion in human RPE cells, effects which could be beneficial in the treatment of AMD. The reduction of inflammatory IL-8 levels was predominantly facilitated by activation of Nrf2 signaling. Further studies will be necessary to fully elucidate the pathways regulated by phloretin and how it exerts its anti-inflammatory effects on IL-6. Phloretin-mediated inhibition of VEGF might involve the phosphatidylinositol 3-kinase (PI3K)/AKT/mammalian target of rapamycin (mTOR) pathway, but this, too, will need further study to confirm [38, 39]. Furthermore, in vitro studies, while an important first step in the study of molecular responses and pathways, can only provide a limited view into the complex mechanisms that underlie human disease. Whether a phloretin-enriched diet would prove beneficial in limiting the incidence or progression of AMD will need to be studied further in animal models and eventually in observational studies in humans.

## Supplementary Information

Below is the link to the electronic supplementary material.

Supplementary file1 (DOCX 95 kb)

## Data Availability

All data generated or analyzed during this study are included in this published article, raw datasets are available from the corresponding author upon reasonable request.
