# Supplementary material for: Phloretin inhibits glucose transport and reduces inflammation in human retinal pigment epithelial cells
Source: Mol Cell Biochem. 2022 Jun 30;478(1):215–27. doi: 10.1007/s11010-022-04504-2 (PMC9836970; doi:10.1007/s11010-022-04504-2)
Supplement: Supplementary file 1 — Supplementary file1 (DOCX 95 kb) [file 11010_2022_4504_MOESM1_ESM.docx]

**Supplementary Information**

*Molecular and Cellular Biochemistry*

**Phloretin inhibits glucose transport and reduces inflammation in human retinal pigment epithelial cells**

Maria Hytti^a^, Johanna Ruuth^a,b^, Iiris Kanerva^a^, Niina Bhattarai^a^, Maria L Pedersen^c^, Carsten U Nielsen^c^, Anu Kauppinen ^a,^*

^a^School of Pharmacy, Department of Health Sciences, University of Eastern Finland, Yliopistonranta 1 C, 70210 Kuopio, Finland

^b^School of Medicine, Department of Health Sciences, University of Eastern Finland, Yliopistonranta 1 C, 70210 Kuopio, Finland

^c^Department of Physics, Chemistry and Pharmacy, University of Southern Denmark, Odense, Denmark

* corresponding author: anu.kauppinen@uef.fi


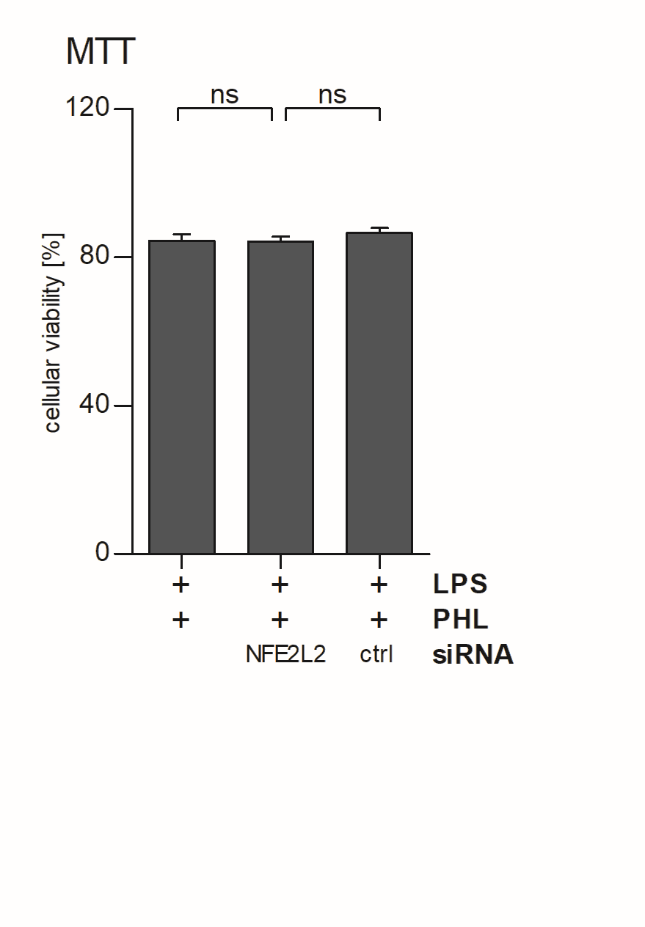


**Supplementary Fig. 1** NFE2L2/Nrf2 knockdown by siRNA transfection or transfection of scrambled control siRNA 24h before exposure to phloretin (PHL) and LPS did not affect cellular viability. Results are combined from three independent experiments with four parallel samples per repetition and are represented as mean ± SEM. ns – not statistically significant, Mann-Whitney U-test
